# Effect of dietary habits on Alzheimer’s disease progression

**DOI:** 10.1002/alz.088759

**Published:** 2025-01-09

**Authors:** Seong Hye Choi, Jee Hyang Jeong, Yangki Minn

**Affiliations:** ^1^ Inha University College of Medicine, Incheon Korea, Republic of (South); ^2^ Ewha Womans University Seoul Hospital, Ewha Womans University College of Medicine, Seoul Korea, Republic of (South); ^3^ Kangnam Sacred Heart Hospital, Seoul Korea, Republic of (South)

## Abstract

**Background:**

Research on the relationship between diet and dementia among Koreans are lacking. This study investigated the association between dietary habits and dementia progression over 3 years in patients with Alzheimer’s disease dementia (ADD).

**Method:**

This study included 705 patients with mild‐to‐moderate ADD. Dietary habits were assessed using the Mini Dietary Assessment Index, comprising 10 questions. Outcome measures included the Clinical Dementia Rating scale‐Sum of Boxes (CDR‐SB), Seoul‐Instrumental Activities of Daily Living, Caregiver‐Administered Neuropsychiatric Inventory (CGA‐NPI), and neuropsychological test battery (NTB) z‐scores, which were evaluated annually over 3 years.

**Result:**

In Q10 (eat all food evenly without being picky), the 3‐year mean differences in CDR‐SB (increases in scores represent worsening) compared to the “rarely” group were –1.86 (95% confidence interval [CI] = –3.64 ∼ –0.09, *P* = 0.039) for the “usually” group and –2.23 (95% CI = –4.40 ∼ –0.06, *P* = 0.044) for the “always” group. In Q7 (add salt or soy sauce to food, when eating), the 3‐year mean differences in CDR‐SB compared to the “always” group were –2.47 (95% CI = –4.70 ∼ –0.24, *P* = 0.030) for the “usually” group and –3.16 (95% CI = –5.36 ∼ –0.96, *P* = 0.005) for the “rarely” group. The “rarely” and “usually” groups in Q7 showed significantly less decline in NTB z‐score and CGA‐NPI compared to the “always” group.

**Conclusion:**

Eating a balanced diet and reducing salt intake were associated with a slower decline in dementia severity, cognition, and behavioral alterations in patients with ADD.

